# Teach different: The CREATE pedagogy for ecology and evolution

**DOI:** 10.1002/ece3.9747

**Published:** 2023-01-16

**Authors:** CJ Lortie

**Affiliations:** ^1^ Department of Biology York University Toronto Ontario Canada

**Keywords:** active teaching, active reading, course design, learning, student work, teaching

## Abstract

Globally, teaching has changed and innovated profoundly in the last 3 years. With novel and more seamless opportunities to use technology and more actively teach visually online or in‐person and to engage in inclusive dialogue within and between groups, we can teach very differently now. An innovation proposed a number of years ago and revisited anew in a recent and novel application to ecology and evolution course offerings, and the CREATE pedagogy or Consider, Read, Elucidate the hypothesis or purpose, Analyze and interpret data or evidence, and Think of the next Experiment is an inspiring framework for unique student work and teaching. CREATE proposes that students engage in active reading through specific exercises that are both creative and heavily anchored in critical thinking when working with publications. Here, the palette of CREATE exercises is expanded further for ecology and evolution and affirms that this approach to student engagement with literature can be highly effective in many courses. Furthermore, the application of this pedagogy dramatically influences and likely enhances how one teaches in a lecture setting as an educator making the content in all modalities more engaging and active.

## Graphical abstract

A recent contribution to Ecology and Evolution proposed a novel pedagogical innovation. In reviewing this paper, it inspired innovations in teaching and student‐led work in two other ecology and evolution course offering. This viewpoint highlights that work and extends it to other potential applications for students and for instructors in how they teach.

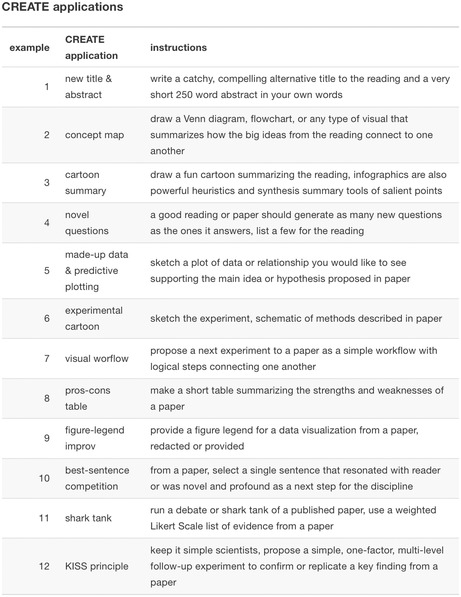

## 1 INTRODUCTION

Science is creative. Reading scientific literature can also be a creative, critical thinking process. Active reading can include highlighting, annotation, and note‐taking (Soto et al., 2019). It can also include asking questions whilst reading—not answering them (Cohen, 1983; Singer, 1978). These approaches to reading and study can increase retention, comprehension, and engagement with scientific ideas (Roy et al., 2021; Toste et al., 2020; Toyokawa et al., 2021). Furthermore, active strategies of deep reading and attention to the methods, results, and data described in peer‐reviewed scientific publications, when reading, can dramatically enhance the reading pattern skills of relatively more junior scientists and students to that akin of expert scientists (Hubbard et al., 2022). However, teaching science and teaching reading in the sciences are not the same, nor likely common.

The teaching philosophy and pedagogy of CREATE are novel and relevant and close this gap, i.e., it bridges teaching science and enables active reading in the same lessons (Hoskins et al., 2007). This framework takes active reading one step further for the sciences. CREATE pedagogy stands for Consider, Read, Elucidate the hypothesis or purpose, Analyze and interpret data or evidence, and Think of the next Experiment (Hoskins et al., 2007). This reading process is creative, active, and phenomenal practice for all professional work. “Consider,” as initially proposed, focuses on the concepts and hypothesis whilst “read” encourages students to read deeply to ensure that they grasp the narrative and the details of the ideas and meaning of key terms. The “elucidate” step of the acronym nudges the learners to define and explain the purpose of the experiment in their own words. “Analyze” engages readers in the process of critical and creative analysis of the meaning of results and their respective presentation in scientific papers. “Think of the next Experiment” is an experimental design‐driven and catalyzes theory development by using the reading as a starting point to forecast and innovate on how to replicate the experiment, address its gaps, and consolidate findings reported in a paper with additional experimentation.

The CREATE pedagogy was first structured around four‐paper sequential reading sets or a module to consolidate concepts and train readers in better predictive thinking (Hoskins et al., 2007). Nonetheless, the pedagogical approach has and can be transposed and adapted into novel implementations that transform lectures into living laboratories wherein we experiment with readings to advance both student comprehension and science.

## 2 PEDAGOGICAL EXAMPLES

A recent paper here in Ecology and Evolution adapted the CREATE framework to both a “Conservation Biology & Biodiversity” and an “Ecology” course (Smith & Paradise, 2022). Students were provided opportunities to use concept maps, extensively annotate results including figures from the paper, draw a schematic of methods, label redacted figure axes, and develop summary tables of key points. It was inspiring and effective in transforming lectures and readings into experimental design thinking and creative opportunities for critical thinking. This is similar to the CREATE pedagogy lessons tested in other courses, but it is the most recent published in a series of implementations in numerous and varied disciplines (Table 1). Two assessment instruments were used, and one was novel and specifically developed for ecology and evolution courses—the Eco‐Evo MAPS (Measuring Achievement and Progress in STEM) tool (Smith & Paradise, 2022; Summers et al., 2018). The very first publication on CREATE proposed and tested this framework to apply active readings to the sciences in “Genetics” and “Cell Biology” with critical thinking as the primary assessment of its efficacy (Hoskins et al., 2007). This original application used a series of 4 related papers (from the same lab) but other examples listed herein often applied the framework to single readings or to a group of readings linked by a theme rather than author. For instance, the next published study (chronologically in terms of the publication date of the paper) tested it in an “Ecology” course using a modified jigsaw approach that split up the CREATE steps into specific exercises and assigned them to different groups of students (Beck, 2019). One paper, many groups. The students then reconvened to draw a global, collective concept map integrating individual group work. In an “Introduction to Genetics” course, all steps were completed by students from concept maps, sketches, and reinterpretation writing exercises, to propose a follow‐up experiment (Krufka et al., 2020). However, in this instance, a grant panel and interview via email of the authors of the paper examined were also tested as a capstone to the CREATE pedagogy process. The workflow in order from concept maps to propose the next experiment was also successfully deployed in “Animal Behavior” (online and in‐person) and in “Biological Psychology” (Bozer, 2022). Collectively, these works found consistent evidence for benefits to students (Table 1, list of assessments reported in papers), thereby supporting this larger movement towards active reading and engagement with published science.

**TABLE 1:** A summary of publications that specifically listed and described the implementation of the CREATE pedagogy for teaching in the sciences. There are other published reading pedagogy studies that similarly leverage active reading principles, and this list is thus specific to the CREATE framework for the sciences. Furthermore, it is a curated list of representative examples from the literature that can spark innovations in teaching ecology. The column year lists the year of the publication, the study title lists the title of the peer‐reviewed publication (see literature cited for full citations), courses list the specific course offerings that used this pedagogy, CREATE applications describes the specific pedagogical tool used, and the assessment column provides the reported assessment instrument used in these publications to examine the relative efficacy of the CREATE pedagogy

Year	Study title	Courses	CREATE applications	Assessment
2007	Selective Use of the Primary Literature Transforms the Classroom Into a Virtual Laboratory	Genetics and cell biology	Student cartoon of the methods of a paper, compare control and experimental result outcomes, predict next experiment, list conclusions of authors and students	Critical thinking test
2019	Integrating Primary Literature in a Lecture Course Using a Modified Version of the C.R.E.A.T.E Approach	Ecology	Jigsaw approach to assign students to groups associated with each CREATE step, concept map, annotate results, schematic of methods, rewrite titles and legends, propose two future studies, and global concept map of all steps as class	Student, peer, and instructor assessments on mastery, understanding, and challenges
2020	A single, narrowly focused CREATE primarily literature module evokes gains in genetics students' self‐efficacy and understanding of the research process	Introduction to genetics	Concept map, paraphrase, sketch, hypothesis in own words, annotate figures and data, propose follow‐up experiment, grant panel, email interview of authors	Test of Scientific Literacy Skills (TOSLS) and Survey of Student Attitudes, Abilities, and Beliefs (SAAB)
2022	A Modified CREATE Approach for Introducing Primary Literature Into Psychological Sciences Courses	Animal behavior (online and in‐person) and biological psychology	Concept map, schematic of methods, rewrite titles and legends, interpret the data, single follow‐up proposed experiment, asynchronous discussion of post about the study	Student survey on perceptions of primary literature, STEM career interest questionnaire, and performance outcomes in terms of grades
2022	Using the CREATE Pedagogy in Ecological Courses	Conservation biology and biodiversity and ecology	Concept map, annotation of results, schematic of methods, prediction plots, label blank axes, key points summary table of paper	Self‐assessment of their learning gains (SALG) instrument, Eco‐Evo MAPS (Measuring Achievement and Progress in STEM)
2022	Teach different: the CREATE pedagogy for Ecology and Evolution	Experimental design for environmental and evolutionary biology and biology for environmental management	Concept maps, predictive plots sketches, schematic of methods, design next experiment	Summative assessments of CREATE tools including a schematic of experiments, predictive plots, and formal adoption as a teaching tool for lessons by an instructor, teaching tool in addition to the student‐led learning tool

However, there are also implications to how we teach and explain via “chalk talks,” and using this framework also implies that we can provide much more diverse and inclusive experiences in what scientific reading, writing, and work looks like and does in scientific lecture halls. There are many other published reading pedagogy studies that similarly leverage active reading principles, but these studies explicitly identified and tested the CREATE framework in the sciences. The specific adaptations in “Conservation Biology & Biodiversity” and “Ecology” (Smith & Paradise, 2022) directly precipitated a deeper professional dive into the CREATE pedagogy and total redesign of two undergraduate course offerings at York University, “Experimental Design for Environmental and Evolutionary Biology” and the offering “Biology for Environmental Management” (Table 1, final row). Success breeds success, and the call to innovate on academic practice in teaching ecology and evolution to promote a growth mindset (Smith & Paradise, 2022) was justified and energizing.

## 3 IMPLICATIONS FOR TEACHING

Teaching the lecture component of some science courses can significantly diverge from the experiential, team‐driven learning that often happens in the associated instructional labs. Ecology and evolution are complex disciplines (Halpern et al., 2020), and the content‐rich material and wide range of theory can consume significant teaching time in lectures. However, the CREATE pedagogy and its published proof‐of‐concept studies listed above suggest that the work students do in reading papers can easily include more active experimental design thinking. This synergy between reading some of the rich content and embracing more pedagogy‐rich teaching in our disciplines (Smith & Paradise, 2022) is a win‐win for students. The palette of CREATE‐infused pedagogical tools is much broader, and likely practiced, than the published instances to date. To that end, here is an expanded but certainly not exhaustive list of applications derived from this active reading and learning framework (Table 2). The concept maps and cartoon summary with novel questions as applications are well tested and enhance student metacognition, i.e., monitoring and control of reading and insights with a focus on process and procedure in addition to content (Soto et al., 2019). Predictive plotting and schematics also firmly support the analyze phase of CREATE and are also commonly reported (Beck, 2019; Smith & Paradise, 2022). The proposed best‐sentence competition is an exercise in evaluating the writing style of a paper alongside its content and messaging. Finally, workflows and other visuals in addition to concept maps and schematics are alternative applications of active reading and learning that can be effective in ecology and evolution and connect with some of the practical research skills we adopt in planning experiments (Lortie et al., 2022). Teaching can thus better align with the active process of doing science.

**TABLE 2:** A list of extended, simplified CREATE applications. Using the CREATE pedagogy and its principles, innovations in application are numerous. This list is not meant to be exhaustive, but it does provide examples of mechanisms to leverage creative, critical thinking principles associated with reading and dissecting peer‐reviewed publications used as readings in science courses such as ecology and evolution. The CREATE application is a simple term to capture the concept. Instructions are one instance of the many mechanisms to communicate the application to students, and the teaching strategies column provides suggested approaches to apply a given tool in ecology and evolution lectures. All of these tools are variations on the initial pedagogy proposed and subsequent examples published describing its success including the most example published here in Ecology and Evolution. For instance, concept maps are commonly used in CREATE courses, but this tool can be explored by students and instructors in other formats such as Venn diagrams or flowcharts. Cartoon summaries are also common, but herein, this tool is advanced by proposing that a selected, common theme such as humor will resonate with students and further engagement

Example	CREATE application	Instructions	Teaching strategies
1	New title and abstract	Write a catchy, compelling alternative title to the reading and a very short 250 word abstract in your own words	Redact sections of paper, collectively ideate, encourage risk and catchy alternatives
2	Concept map	Draw a Venn diagram, flowchart, or any type of visual that summarizes how the big ideas from the reading connect to one another	Provide an example from your discipline or recent paper concept mapped out that you or your team have grappled with, or provide a conventional concept map for the reading and task students with transforming to Venn diagram or flowchart
3	Cartoon summary	Draw a fun cartoon summarizing the reading, infographics are also powerful heuristics and synthesis summary tools of salient points, choose a theme	Numerous resources online for inspiration including https://phdcomics.com/ or xkcd.com and infographics are common in ecology, evolution, and the environmental sciences, if needed, many ten simple rules papers describing effective scientific communication principles
4	Novel questions	A good reading or paper should generate as many new questions as the ones it answers, list a few for the reading	Ideate collectively, discuss a typology for scientific questions
5	Made‐up data and predictive plotting	Sketch a plot of data or relationship you would like to see supporting the main idea or hypothesis proposed in paper	Discuss illustrative data and ideal scenarios as a starting point for support for hypotheses, sketch out specific predictions as stepping stones to support
6	Experimental cartoon	Sketch the experiment, schematic of methods described in paper	Provide a sketch of the methods of a paper, how the work was done, not the outcomes
7	Visual workflow	Propose a next experiment to a paper as a simple workflow with logical steps connecting one another	Query visual workflows online and explore images that inspire and link to the specific topic
8	Pros‐cons table	Make a short table summarizing the strengths and weaknesses of a paper	Apply this approach to methods, results, and implications, encourage the view that there is no perfect experiment and reminders learners that science is a process
9	Figure‐legend improv	Provide a figure legend for data visualization from a paper, redacted or provided	Provide only the figure, curate a small collection of examples for the topic, provide actual figure legends post hoc and discuss consilience
10	Best‐sentence competition	From a paper, select a single sentence that resonated with a reader or was novel and profound as a next step for the discipline	Provide examples of sentences that shifted your view on a topic from papers, it can be funny, honest, transparent, transformative, profound, incorrect, or an implication
11	Shark tank	Run a debate or shark tank of a published paper, use a weighted Likert Scale list of evidence from a paper	Split readers into groups, assign them to rank evidence on a Likert Scale in the strengths and weaknesses, consider a brief debate or tallying of scores to informally rank papers that would successfully secure funding for a next experiment
12	KISS principle	Keep it simple scientists, propose a simple, one‐factor, multilevel follow‐up experiment to confirm or replicate a key finding from a paper	Science needs replication, including ecology and evolution, identify a main finding, then get creative and design simple experiments that can replicate the key finding

We need to be as inclusive and diverse as possible in our offerings of tools to promote comprehension of our disciplines and the inherent complexity in nature that we work so rigorously to describe through scientific inquiry. Finally, the win‐win of these tools does not end with the students. Teach different too. In planning these opportunities for Experimental Design for Environmental and Evolutionary Biology" and "Biology for Environmental Management," it rapidly became evident that extemporaneous teaching with these same tools on the board (or digitally for remote teaching using any open notepad tool) made teaching more active. In addition to an oral presentation with slide decks, putting these applications into practice will change how you lecture. The same tools that you propose the students adopt to become active readers can help one become a more active teacher. The schematics, predictive plots, flowcharts, and concept maps—sketched out as one teaches—honed our classwide critical thinking skills as learners and co‐learners in the lectures for these two‐course offerings. Practice makes practice, and these specific science practice skills (Smith & Paradise, 2022) are done openly and often imperfectly as an instructor can shift an ecology and evolution lecture in a classroom towards a shared community that functions more like a lab or team science endeavor.

## AUTHOR CONTRIBUTIONS

**CJ Lortie:** Conceptualization (equal); methodology (equal).

## CONFLICT OF INTEREST

The author declares no conflict of interest financially or otherwise.

### OPEN RESEARCH BADGES

This article has earned Open Data and Open Materials badges. Data and materials are available at https://doi.org/10.6084/m9.figshare.21691757.v1 and https://doi.org/10.6084/m9.figshare.21691757.v1.

## Data Availability

There are no data directly associated with this viewpoint.
